# Supplementary material for: Structural basis of gating modulation of Kv4 channel complexes
Source: Nature. 2021 Sep 22;599(7883):158–64. doi: 10.1038/s41586-021-03935-z (PMC8566240; doi:10.1038/s41586-021-03935-z)
Supplement: Supplementary file 1 — This file contains Supplementary Discussion, Supplementary Figures 1–12 and Supplementary Table 1. [file 41586_2021_3935_MOESM1_ESM.pdf]

nature portfolio

https://doi.org/10.1038/s41586-021-03935-z

Supplementary information

Structural basis of gating modulation of Kv4 channel complexes

In the format provided by the authors and unedited

Supplementary Discussion

Modulation by KChIP1

KChIPs reportedly prevent OSI and accelerate CSI and recovery from inactivation (Fig. 3, Extended Data Fig. 7a, Supplementary Fig. 5) (11,24,36). The structural comparison between Kv4.2 alone and Kv4.2-KChIP1 complex provides insight into how KChIPs modulate gating of Kv4s. In the Kv4.2-KChIP1 complex, KChIP1s bind and sequester the both N-terminal inactivation ball and C-terminus (amino acids 472-495) of Kv4.2, which would therefore result in preventing N-type inactivation. Moreover, while S6 gating helices adopt more flexible conformation with weaker interaction with T1-S1 linkers in the structure of Kv4.2 alone, KChIP1 stabilizes these structures and enhances their interactions in the structure of Kv4.2-KChIP1. These structural changes mediated by KChIPs, together with the following three observations and reports, might explain how KChIPs accelerate S6 gating of Kv4s including CSI and recovery from inactivation. First, one KChIP1 stabilizes S6 conformation as well as N-terminus from the neighboring subunit of Kv4.2. Second, one KChIP1 also interacts with two T1 domains from neighboring subunits (Fig. 2b) (26,27). Third, previous functional studies have suggested that the T1-S1 linker of Kv4 dodecameric channels undergoes major conformational shifts tightly coupled to movements of the S6 tail upon binding with KChIP1 (49,50), although we do not know what T1 conformation change is. Together, these structural features mediated by KChIP1 may allow synchronized and accelerated S6 gating to enable fast CSI and recovery (Extended Data Fig. 13a).

Modulation by DPP6

DPP6S reportedly accelerates the activation, inactivation, and recovery of K4 channels (39). In the Kv4.2-DPP6S complexes, the single-spanning transmembrane helix of DPP6S apparently stabilizes the structure of S1 and S2 helices because it simultaneously interacts with the lower half of S1 and the upper half of S2 (Fig. 4a, b). DPP6S reportedly accelerates both the outward and inward movements of the Kv4.2 gating charge upon depolarization and repolarization, respectively (28). Among the hypotheses to explain the voltage dependency in voltage-gated channels, the hypothesis that S4 slides on the surface formed by S1 and S2 depending on the membrane potential might be most likely (13). Therefore, the stabilization of the S1-S2 conformation may facilitate the movement of the S4 helices upon depolarization and repolarization, which could explain the fast kinetics of activation and recovery from the closed inactivated state (Extended Data Fig.13b).

Previous studies suggest that DPP6S accelerates both OSI and CSI of Kv4s (Extended Data Fig.13b) (40,51). The acceleration of open-state inactivation by DPP6S could involve the N-terminal intracellular domain of DPP6S and N-terminus of Kv4s (40), however, both regions are disordered in the structure of Kv4.2-DPP6S and further investigations are required. Previous studies suggest that the dynamic interaction of S4-S5 linker and S6 gate is the molecular basis of closed state inactivation (12, 22). Therefore, the acceleration of CSI by DPP6S could be, at least in part, attributed to the accelerated conformational change of S4 as discussed above (Extended Data Fig.13b).

Modulation in the Kv4 macromolecular ternary complex

Native Kv4s form macromolecular ternary complex with KChIPs and DPPs. The structure of Kv4.2-DPP6S-KChIP1 dodecameric complex (Fig. 1a) supports the additive contribution of KChIPs and DPPs to the modulation of Kv4s in the ternary complex. KChIP1 and DPP6S interact with distinct structures of Kv4.2 to modulate its gating kinetics in different manners (Fig. 1a, Fig. 2, Fig. 4a). In addition, KChIP1 and DPP6S do not interact with each other. Overall, the modulatory mechanisms of Kv4.2 by KChIP1 and DPP6S are different, and therefore, native Kv4 channels form ternary macromolecular complexes with both KChIPs and DPPs to exhibit eliminated OSI, accelerated CSI, and fast recovery rate from CSI (Extended Data Fig.13c) (5). Structurally mechanistic elucidations of CSI will further clarify the mechanisms of modulation by KChIPs and DPPs.

Insight into closed-state inactivation of Kv4.2

The structural correlates of Kv4 in the closed state inactivation (CSI) remain unknown. Previous studies have proposed that the interaction between the S4-S5 linker and S6 in Kv4s, which couples the S4 movement to S6 gating in Kv1, might be lost following the upshifted movement of S4 during depolarization (Extended Data Fig. 1b) (12,21,22). Indeed, the amino acid sequences of Kv4 around the S4-S5 linker and S6 on the intracellular side are unique among the *Shaker*-related Kv subfamilies (Kv1-Kv4) (Supplementary Fig. 12a), and mutations of these regions affect the closed state inactivation kinetics of Kv4 (Supplementary Fig. 12b) (21,22). In addition, the open conformation of Kv4.2 complexes revealed several Kv4-specific residues involved in the

intra-subunit interactions between the S4-S5 linker and S6, as well as the inter-subunit interactions between the S4-S5 linker and S5 (Supplementary Fig. 12a, c, d). Further study of this “pre-closing” conformation may lead to elucidating the mechanism of CSI. Together, future structural studies of the resting and closed inactivated states will provide more mechanistic insights into Kv4 channel gating, closed state inactivation, and modulation by auxiliary subunits.

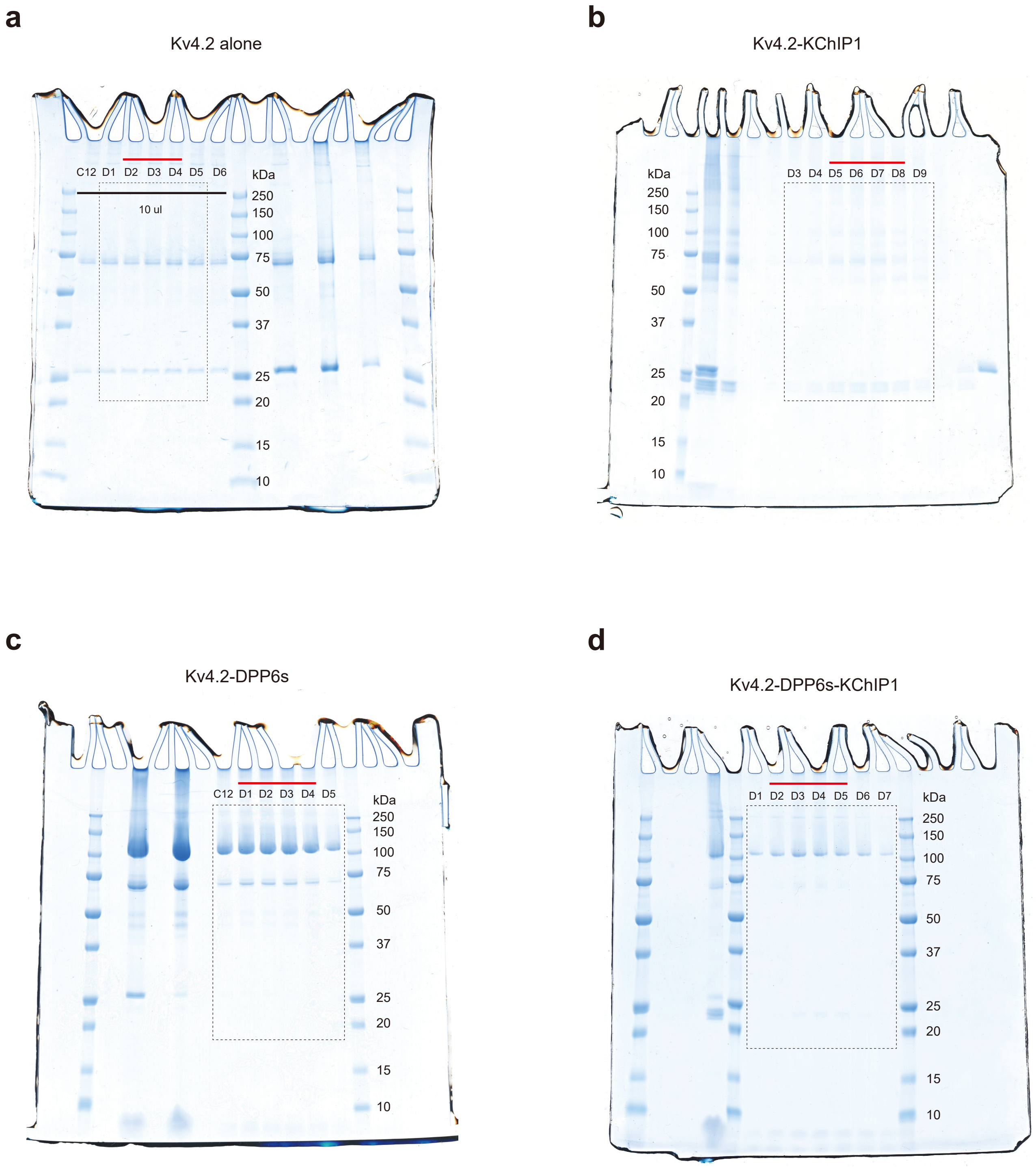

Supplementary Fig. 1. Raw gel images for Extended Data Fig. 2.

a. Raw gel image of Kv4.2 alone after SEC. Fractions surrounded by dotted box are shown in Extended Data Fig. 2c. Fractions indicated by red bars were pooled for cryo-EM grid preparation.

b. Raw gel image of Kv4.2-KChIP1 complex after SEC. Fractions surrounded by dotted box are shown in Extended Data Fig. 2d. Fractions indicated by red bars were pooled for cryo-EM grid preparation.

c. Raw gel image of Kv4.2-DPP6S complex after SEC. Fractions surrounded by dotted box are shown in Extended Data Fig. 2e. Fractions indicated by red bars were pooled for cryo-EM grid preparation.

d. Raw gel image of Kv4.2-DPP6S-KChIP1 after SEC. Fractions surrounded by dotted box are shown in Extended Data Fig. 2f. Fractions indicated by red bars were pooled for cryo-EM grid preparation.

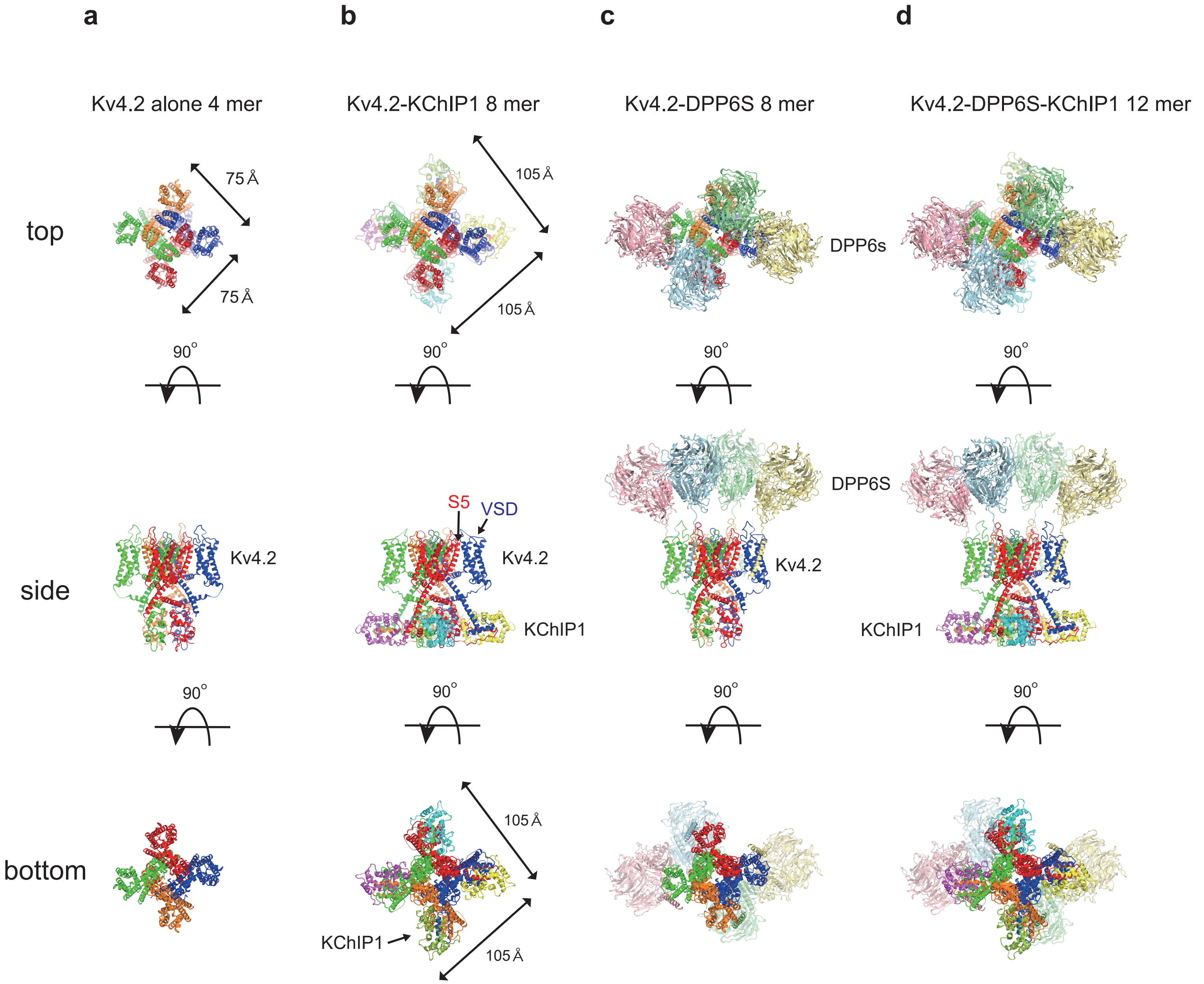

Supplementary Fig. 2. Overall structures of Kv4.2 alone, Kv4.2-KChIP1, Kv4.2-DPP6S, and Kv4.2-DPP6S-KChIP1 complexes.

a. The structure of Kv4.2 alone from the top, side, and bottom views. Each subunit is illustrated in a different color.

b. The structure of Kv4.2-KChIP1 complex from the top, side, and bottom views. Each subunit is illustrated in a different color. The side view shows that the S1-S4 voltage sensor domain (VSD) (blue) interacts with S5 of the pore domain from the neighboring subunit (red).

c. The structure of Kv4.2-DPP6S complex from the top, side, and bottom views. Each subunit is illustrated in a different color.

d. The structure of Kv4.2-DPP6S-KChIP1 complex from the top, side, and bottom views. Each subunit is illustrated in a different color.

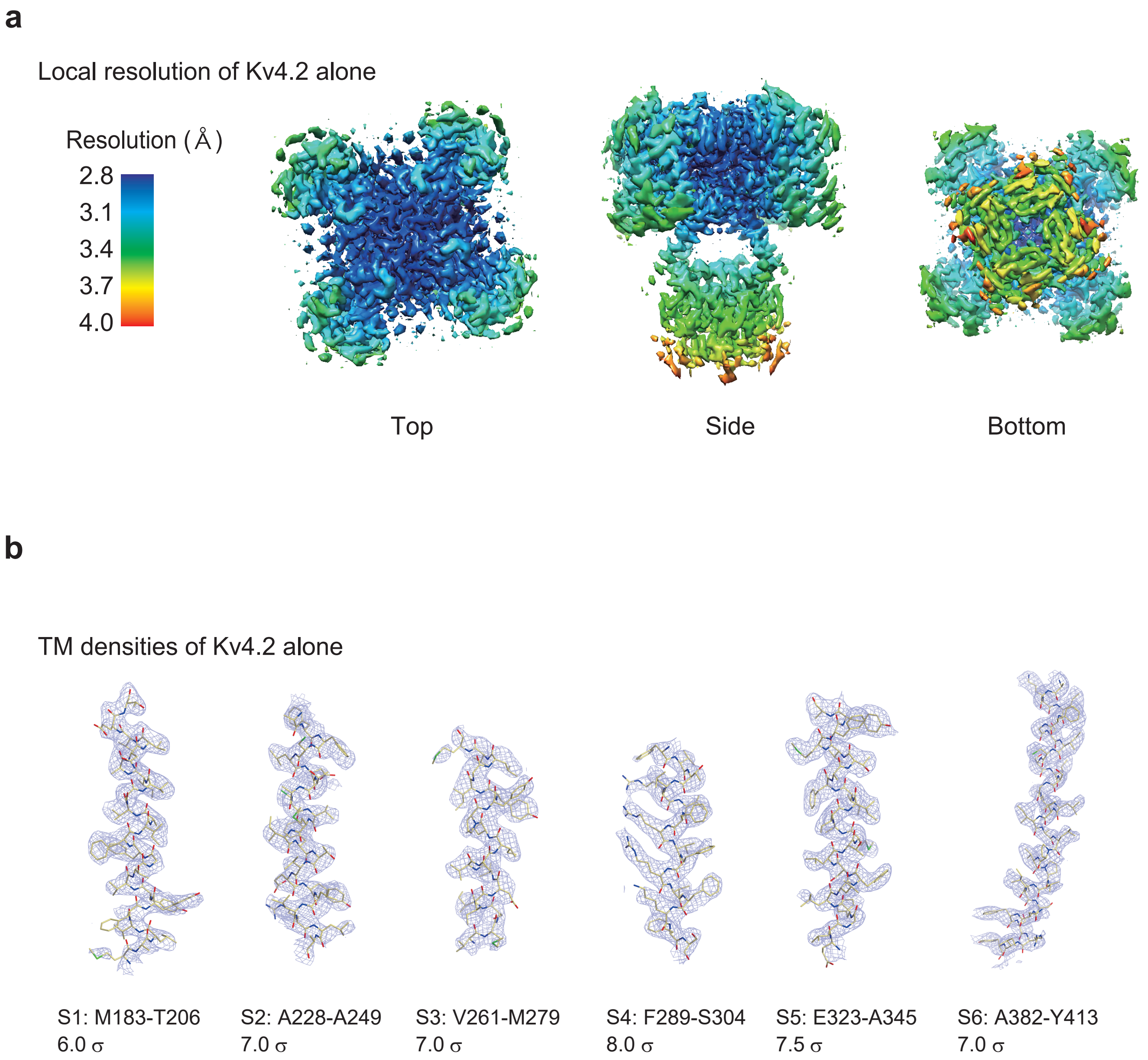

Supplementary Fig. 3. Density map and model building of Kv4.2 alone related to Extended Data Fig. 3.

a. Local resolution of Kv4.2 alone.

b. TM density and model building of Kv4.2 alone.

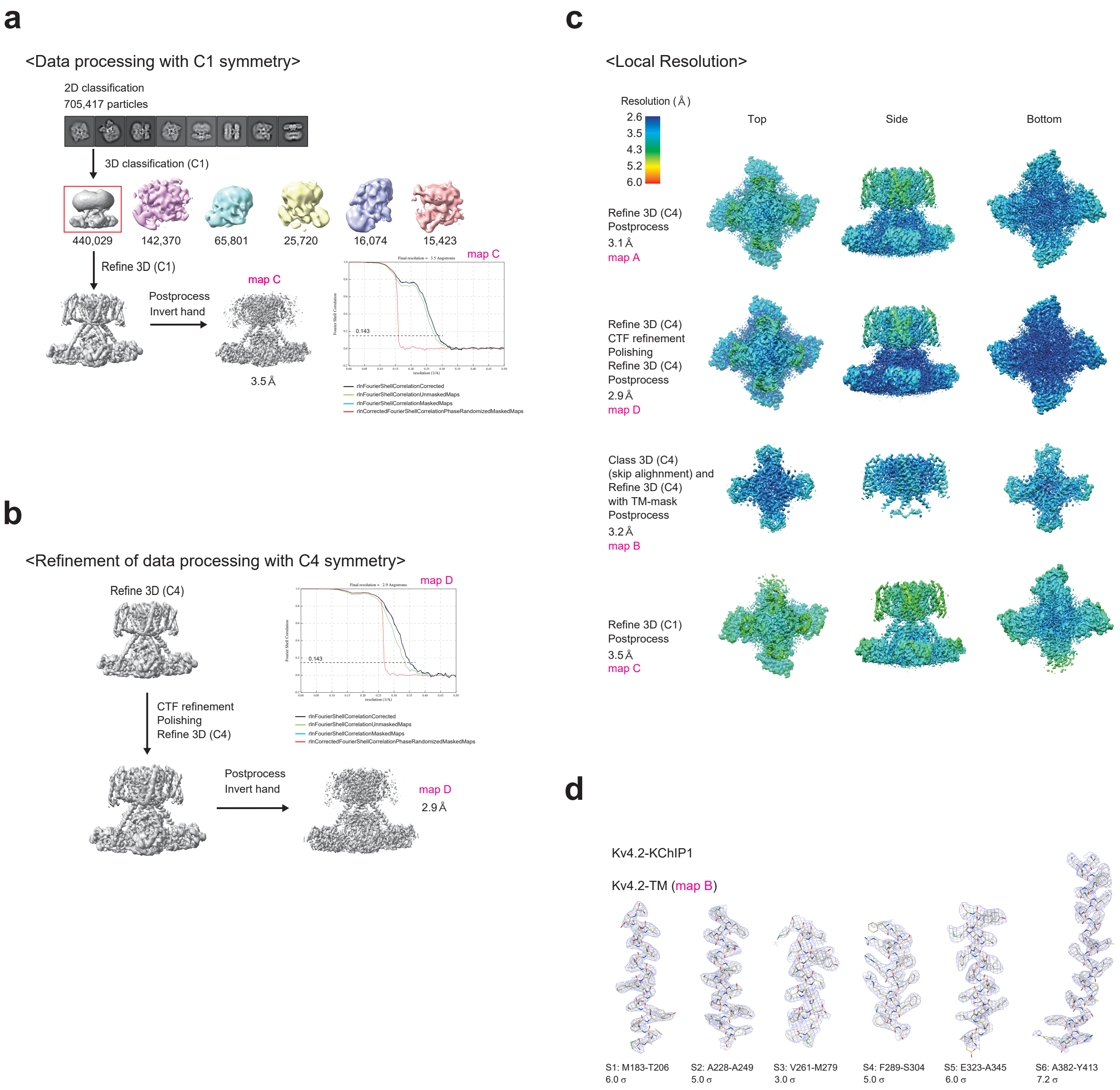

Supplementary Fig. 4. Density map and model building of Kv4.2-KChIP1 complex related to Extended Data Fig. 4.

a. The structure was determined by data processing with C1 symmetry, resulting in lower overall resolution (3.5 Å) than that with C4 symmetry imposed (3.1 Å) (map A in Extended Data Fig. 4).

b. The density map with overall resolution of 2.9 Å was obtained after CTF refinement and polishing.

c. Local resolution of each density map.

d. TM density and model building of map B.

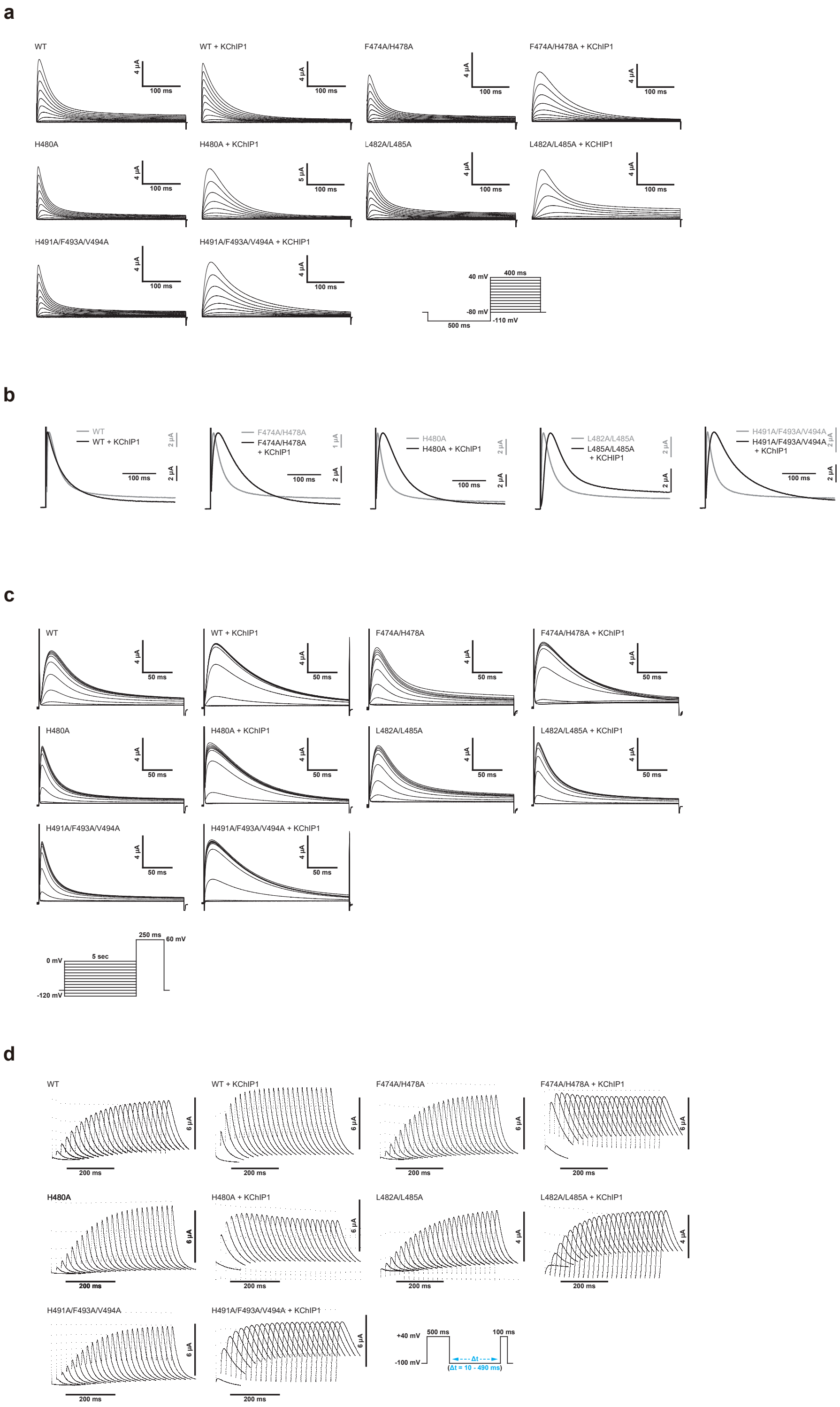

Supplementary Fig.5. Influence of Kv4.2-KChIP1 interface mutations on KChIP1 modulation.

a. Representative current traces elicited by depolarization for WT and each mutant with or without KChIP1. The holding potential was -80 mV. After 500 ms of hyperpolarization at -110 mV to remove inactivation, currents were elicited by 400 ms test pulses to membrane potentials from -80 to 40 mV with 10 mV increments (inset) (n = 8 independent experiments).

b. Normalized and superposed current traces of WT with (black) or without (gray) KChIP1, and each mutant with (black) or without (gray) KChIP1 elicited by test pulses of 40 mV for the qualitative comparisons of inactivation kinetics (n = 8 independent experiments).

c. Representative current traces elicited by prepulse inactivation protocol for WT and each mutant with or without KChIP1. The holding potential was -100 mV. After 5 sec of prepulses from -120 mV to 0 mV with 10 mV increments, currents were elicited by 250 ms test pulses at 60 mV (inset). Only the current traces elicited by the test pulses are presented (n = 8 independent experiments).

d. The recovery from inactivation for WT and each mutant with or without KChIP1. The currents were elicited by a two-pulse protocol (inset) using the prepulses (500 ms) and the test pulses (100 ms) at 40 mV with an interpulse interval of the duration from 10 to 490 ms at -100 mV. Only the current traces elicited by the test pulses are presented (n = 8 independent experiments).

<Local Resolution>

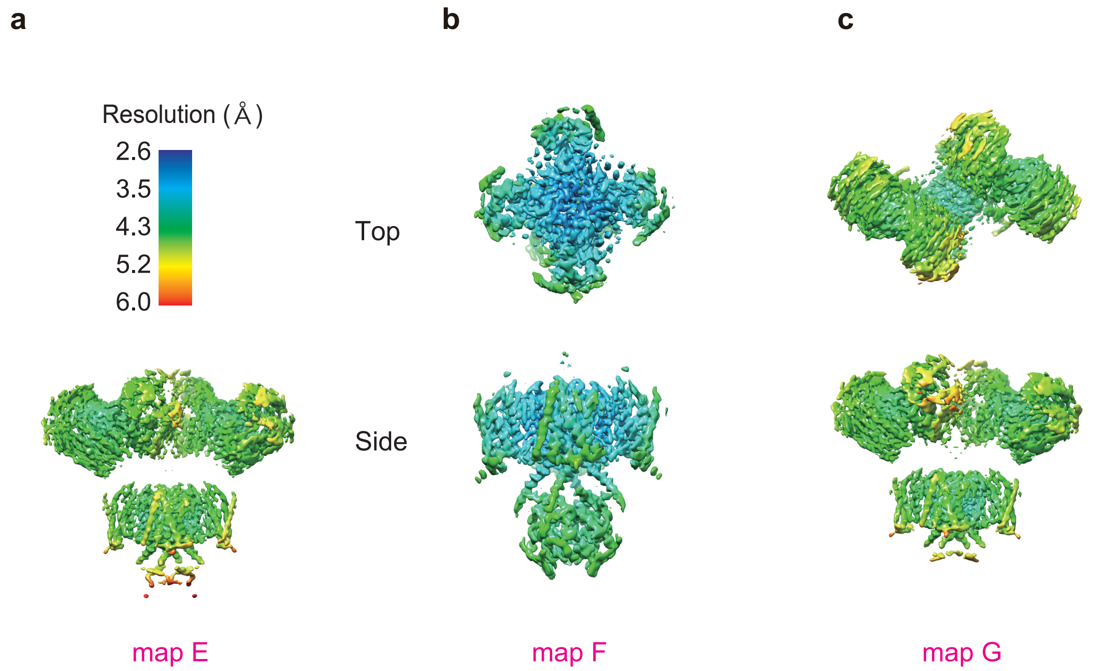

<TM density>

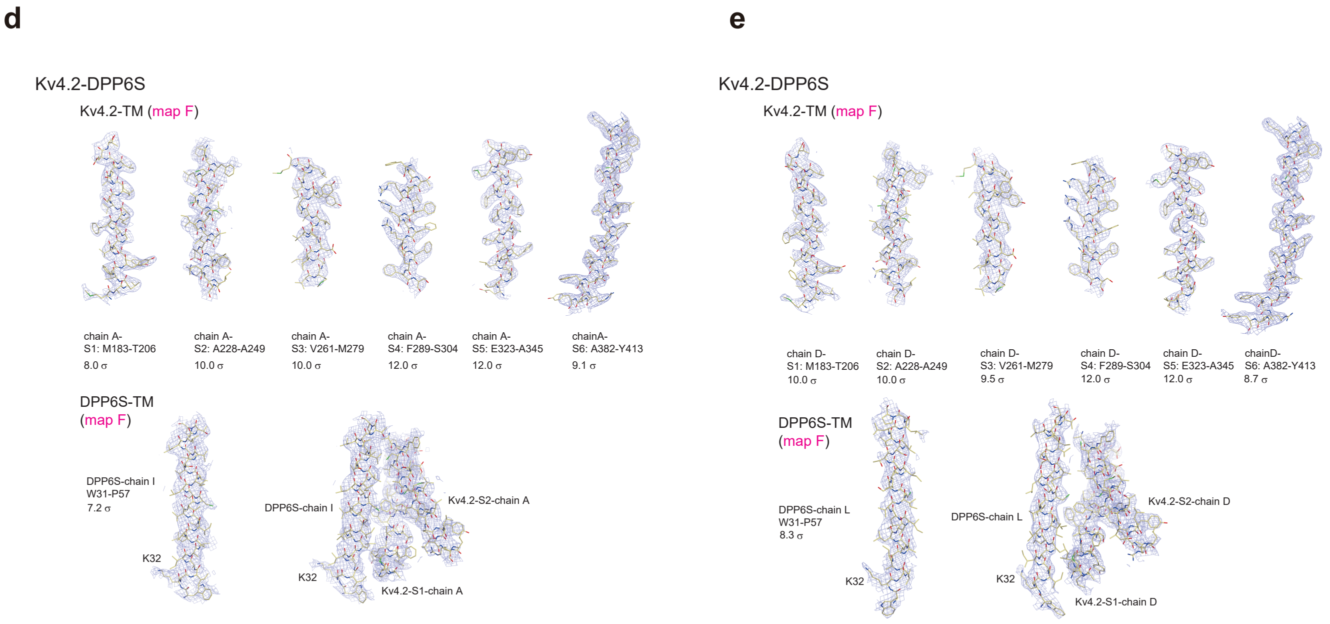

Supplementary Fig. 6. Density map and model building of Kv4.2-DPP6S complex related to Extended Data Fig. 8.

a. Local resolution of whole density map (map E).

b. Local resolution of TM and intracellular parts with focused refinement (map F).

c. Local resolution of TM and extracellular parts with focused refinement (map G).

d, e. TM density and model building of map F.

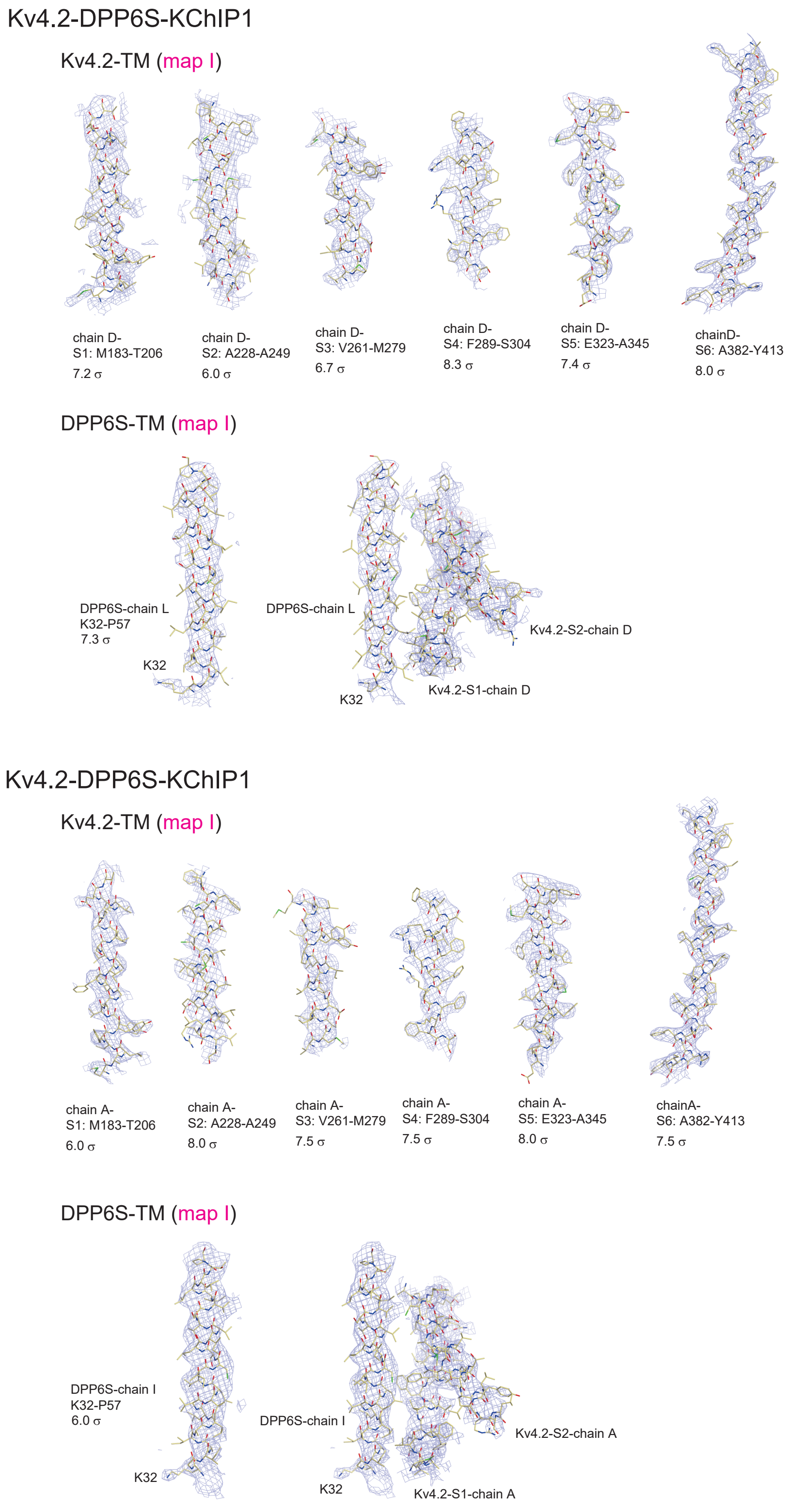

Supplementary Fig. 7. TM density map and model building of Kv4.2-DPP6S-KChIP1 complex related to Extended Data Fig. 9.

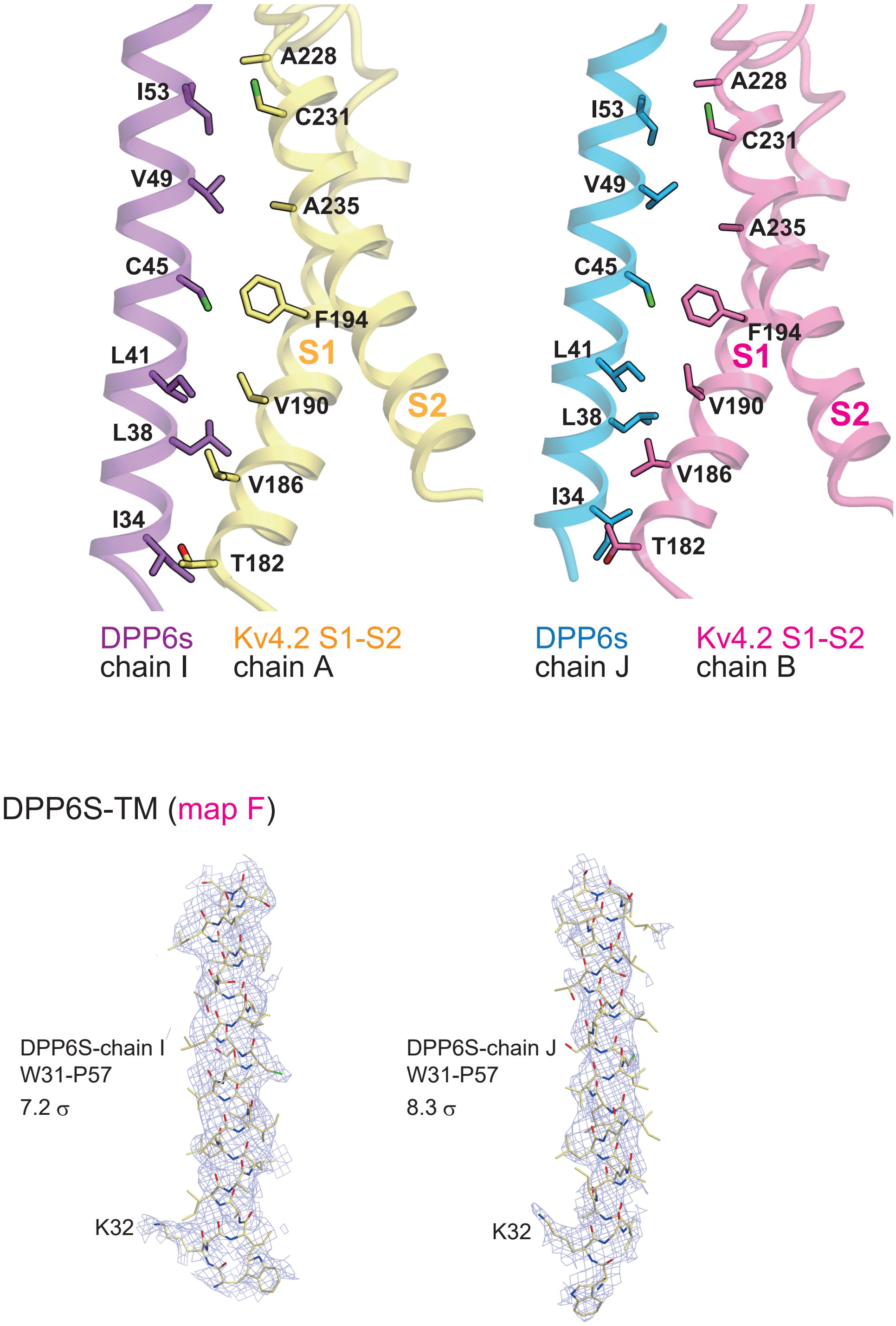

Supplementary Fig. 8. Kv4.2-DPP6S interaction in the transmembrane region. Residues in the DPP6S transmembrane domain at two out of four interaction sites with Kv4.2 are shown from the Kv4.2-DPP6S complex with C2 symmetry imposed (upper). Model building of the DPP6S transmembrane part was performed with the help of a lysine-like density at K32 (bottom).

a

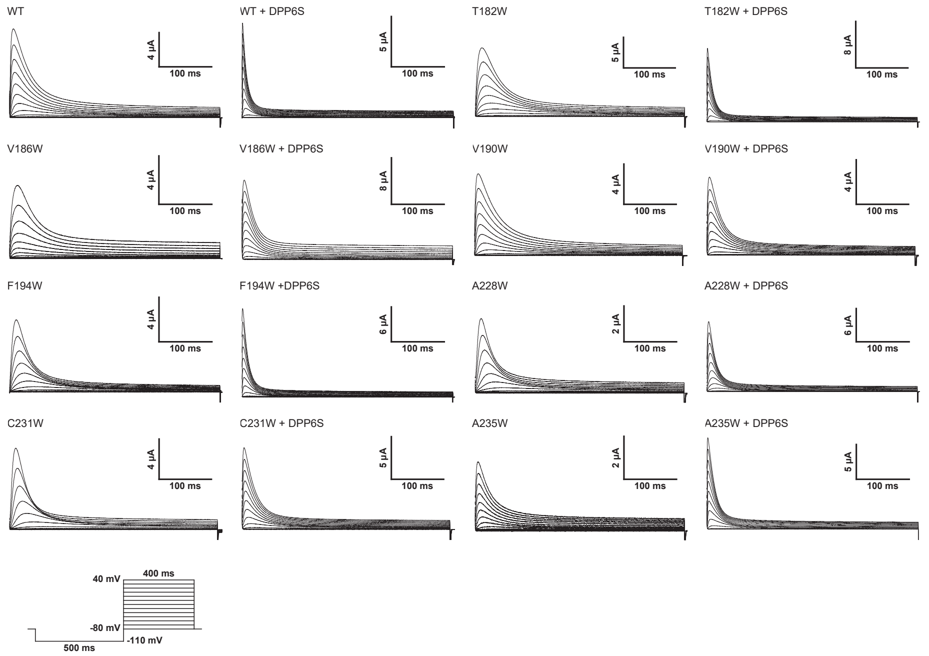

b

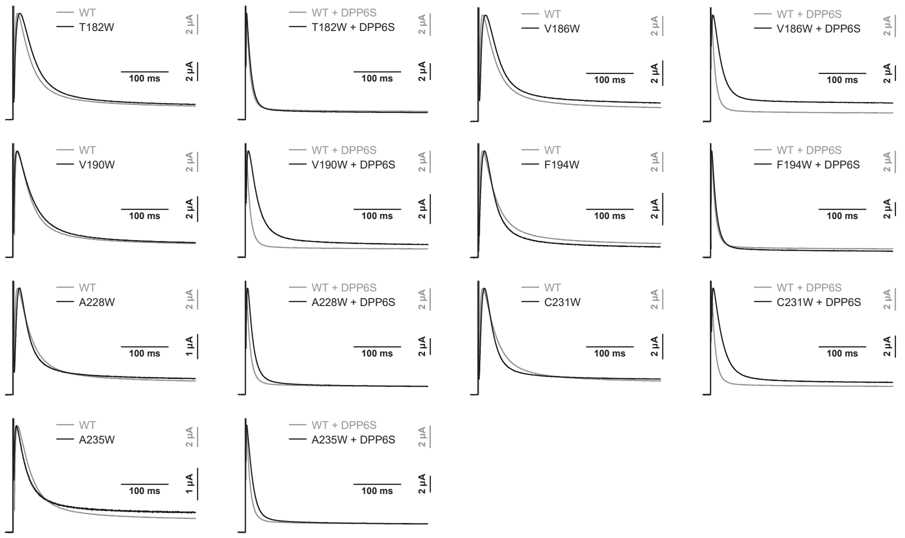

c

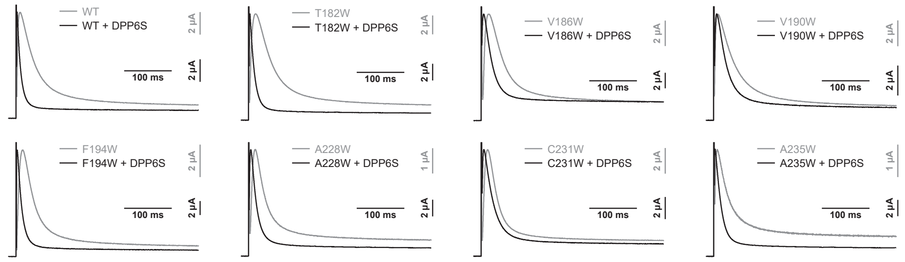

Supplementary Fig. 9. Influence of Kv4.2-DPP6S interface mutations on DPP6S modulation: current traces.

a. Representative current traces elicited by depolarization for WT and each mutant with or without DPP6S. The holding potential was -80 mV. After 500 ms of hyperpolarization at -110 mV to remove inactivation, currents were elicited by 400 ms test pulses to membrane potentials from -80 to 40 mV with 10 mV increments (inset) (n = 8 independent experiments).

b. Normalized and superposed current traces of WT (gray) and each mutant (black) (left), and WT with DPP6S (gray) and each mutant with DPP6S (black) (right) elicited by test pulses of 40 mV for the qualitative comparisons of inactivation kinetics (n = 8 independent experiments).

c. Normalized and superposed current traces of WT with (black) or without (gray) DPP6S, and each mutant with (black) or without (gray) DPP6S elicited by test pulses of 40 mV for the qualitative comparisons of inactivation kinetics (n = 8 independent experiments).

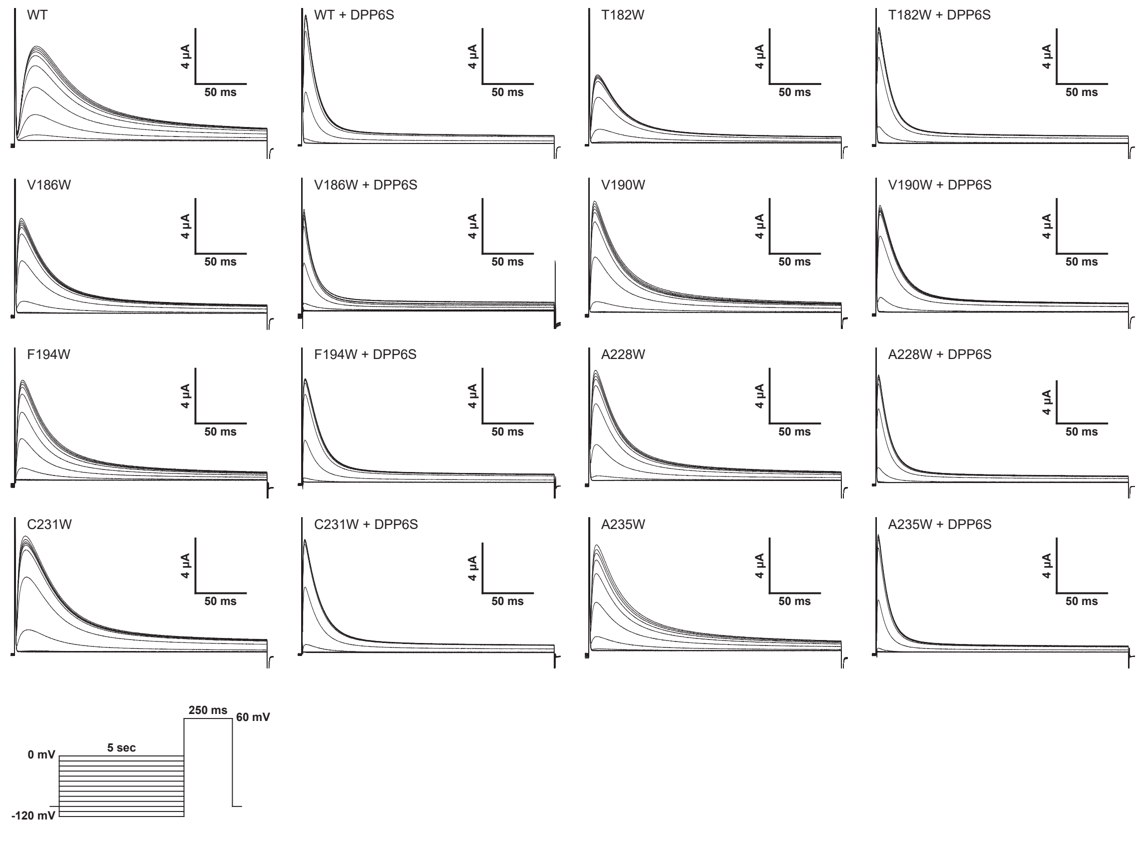

Supplementary Fig. 10. Influence of Kv4.2-DPP6S interface mutations on DPP6S modulation: voltage-dependent inactivation.

Representative current traces elicited by prepulse inactivation protocol for WT and each mutant with or without DPP6S. The holding potential was -100 mV. After 5 sec of prepulses from -120 mV to 0 mV with 10 mV increments, currents were elicited by 250 ms test pulses at 60 mV (inset). Only the current traces elicited by the test pulses are presented (n = 8 independent experiments).

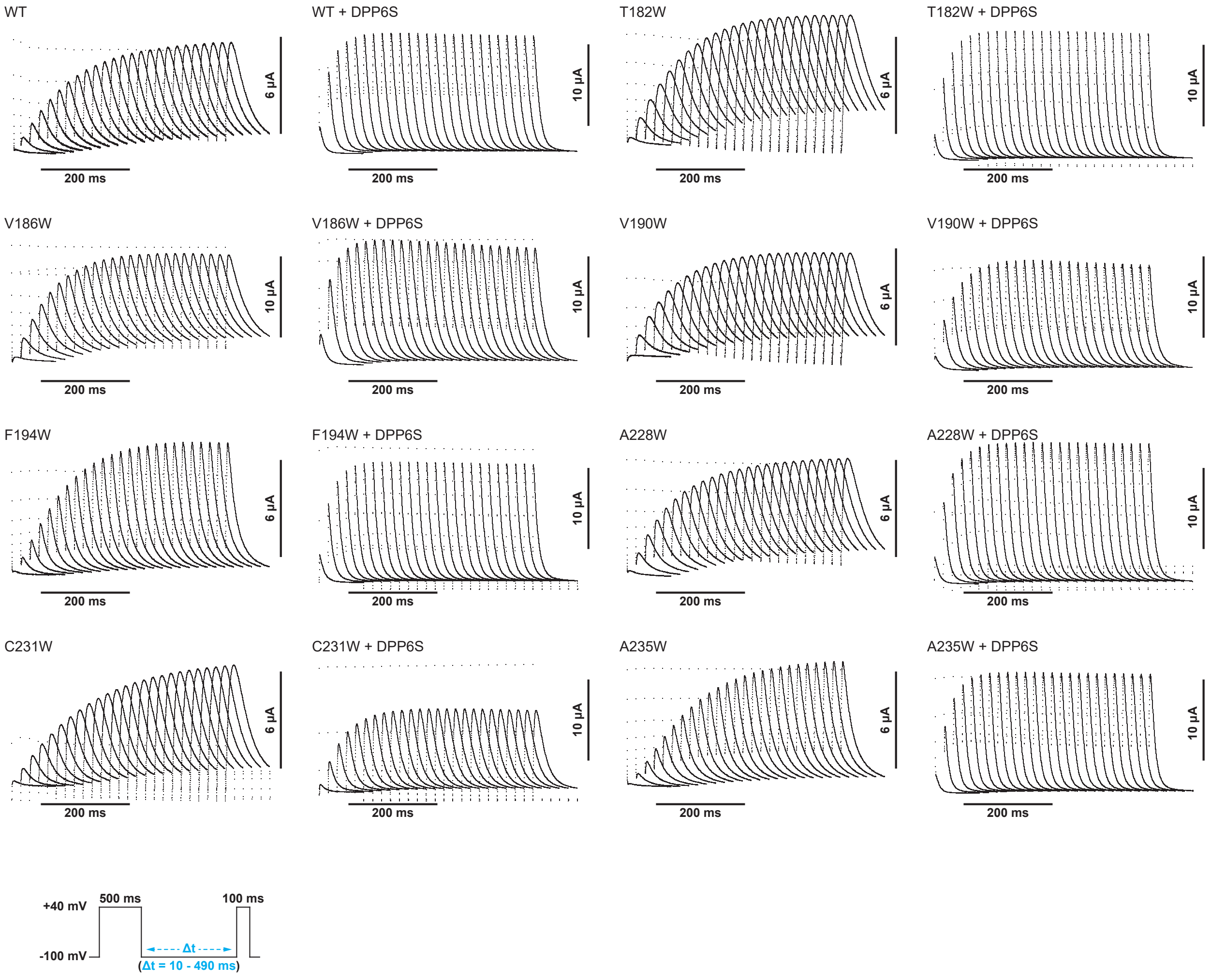

Supplementary Fig. 11. Influence of Kv4.2-DPP6S interface mutations on DPP6S modulation: recovery from inactivation.

The recovery from inactivation for WT and each mutant with or without DPP6S. The currents were elicited by a two-pulse protocol (inset) using the prepulses (500 ms) and the test pulses (100 ms) at 40 mV with an interpulse interval of the duration from 10 to 490 ms at -100 mV. Only the current traces elicited by the test pulses are presented (n = 8 independent experiments).

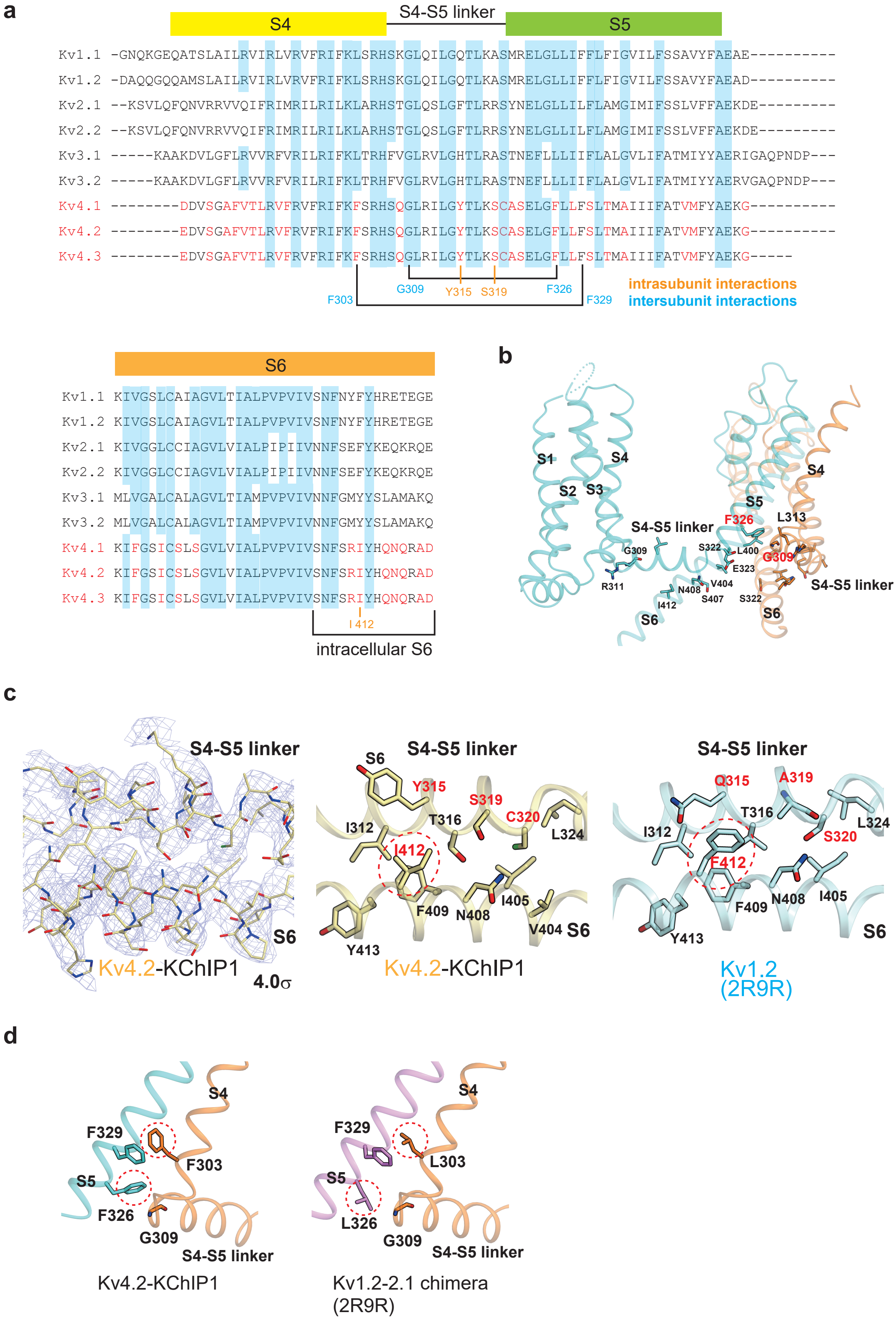

Supplementary Fig. 12. Structural analysis of the closed state inactivation of Kv4.2.

a. Amino acid sequence alignment of S4, S5, and S6 from Kv1 to Kv4. The Kv4 specific residues are colored red and distributed throughout these regions. Specifically, Y315, S319, and I412 are involved in the intra-subunit interactions of the S4-S5 linker and S6. In addition, F303 and F326 are involved in the intersubunit interactions of the S4/S4-S5 linker and S5. See also (c) and (d) for details.

b. Mapping of residues involved in the closed state inactivation of Kv4, identified by Wollberg and Bähring (Biophys J, 110, 157-175, 2016). G309 and F326 are involved in the intersubunit interactions and are highlighted in red.

c. Comparison of intra-subunit interactions between the S4-S5 linker and S6 in Kv1.2 and Kv4.2 (middle, right). A density map of Kv4.2-KChIP1 in this region is also shown (left). Specific residues in each Kv subfamily are shown in red. Especially, the substitution of F412 in Kv1.2 to I412 in Kv4 may result in weaker interactions with the S4-S5 linker (Extended Data Fig. 1, 13).

d. Comparison of the intersubunit interactions between the S4/S4-S5 linker and S5 in Kv1.2 and Kv4.2. Especially, the substitution of L326 in Kv1.2 to F326 in Kv4.2 may result in stronger interactions, which may stabilize the conformation of the S4-S5 linker to facilitate the (partial) detachment of S6 (Extended Data Fig. 1, 13).

Condition	Slow compoment fraction$_{@40\ mV}$	$\tau_{slow@40\ mV}$ (ms)	$\tau_{fast@40\ mV}$ (ms)	Slow compoment fraction$_{@30\ mV}$	$\tau_{slow@30\ mV}$ (ms)	$\tau_{fast@30\ mV}$ (ms)	Slow compoment fraction$_{@20\ mV}$	$\tau_{slow@20\ mV}$ (ms)	$\tau_{fast@20\ mV}$ (ms)	Slow compoment fraction$_{@10\ mV}$	$\tau_{slow@10\ mV}$ (ms)	$\tau_{fast@10\ mV}$ (ms)	Slow compoment fraction$_{@0\ mV}$	$\tau_{slow@0\ mV}$ (ms)	$\tau_{fast@0\ mV}$ (ms)
**α subunit alone**															
Kv4.2 WT	0.19 ± 0.03	168.8 ± 8.8	29.0 ± 1.4	0.19 ± 0.03	185.8 ± 14.7	31.1 ± 1.6	0.20 ± 0.02	235.4 ± 45.9	33.8 ± 1.7	0.22 ± 0.03	289.6 ± 65.2	37.1 ± 1.8	0.24 ± 0.04	398.3 ± 102.6	41.9 ± 2.3
Kv4.2 T182W	0.18 ± 0.03	200.7 ± 22.7	32.5 ± 3.4	0.18 ± 0.03	330.8 ± 62.7	36.5 ± 4.3	0.20 ± 0.03	318.3 ± 40.0	39.3 ± 4.0	0.23 ± 0.04	449.0 ± 51.5	46.5 ± 5.3	0.30 ± 0.05	462.4 ± 69.6	54.6 ± 5.1
Kv4.2 V186W	0.22 ± 0.03	240.5 ± 25.5	34.9 ± 3.1	0.24 ± 0.04	462.1 ± 86.8	39.4 ± 3.7	0.25 ± 0.04	569.3 ± 64.3	43.9 ± 3.8	0.30 ± 0.05	627.3 ± 55.0	49.7 ± 4.2	0.31 ± 0.06	539.7 ± 49.0	58.8 ± 4.6
Kv4.2 V190W	0.21 ± 0.02	180.9 ± 13.6	31.0 ± 1.1	0.19 ± 0.02	306.5 ± 56.6	35.0 ± 1.4	0.21 ± 0.03	273.7 ± 46.0	37.7 ± 1.8	0.26 ± 0.04	436.9 ± 120.2	42.1 ± 3.0	0.31 ± 0.06	376.5 ± 145.7	47.3 ± 5.1
Kv4.2 F194W	0.20 ± 0.04	145.4 ± 11.4	26.2 ± 1.6	0.17 ± 0.04	258.7 ± 43.6	30.9 ± 2.0	0.18 ± 0.05	307.0 ± 60.1	33.9 ± 2.3	0.20 ± 0.05	393.6 ± 48.0	38.4 ± 2.5	0.22 ± 0.06	367.9 ± 70.6	44.2 ± 3.0
Kv4.2 A228W	0.18 ± 0.03	152.3 ± 13.1	25.9 ± 2.2	0.17 ± 0.03	197.4 ± 24.5	28.7 ± 2.4	0.18 ± 0.04	342.5 ± 72.0	32.3 ± 2.7	0.19 ± 0.04	442.0 ± 68.8	36.6 ± 2.2	0.23 ± 0.04	685.2 ± 69.3	43.2 ± 1.8
Kv4.2 C231W	0.18 ± 0.08	158.4 ± 42.4	36.8 ± 6.9	0.19 ± 0.06	193.3 ± 51.6	41.0 ± 7.5	0.20 ± 0.06	289.3 ± 76.1	48.9 ± 8.4	0.20 ± 0.06	424.1 ± 110.7	62.3 ± 10.9	0.23 ± 0.07	430.9 ± 107.4	80.4 ± 13.6
Kv4.2 A235W	0.22 ± 0.04	107.8 ± 2.7	21.7 ± 0.6	0.20 ± 0.04	117.6 ± 4.2	23.2 ± 0.8	0.18 ± 0.04	130.7 ± 6.6	24.7 ± 1.0	0.18 ± 0.04	144.9 ± 9.2	26.1 ± 1.1	0.16 ± 0.04	171.2 ± 13.8	28.7 ± 1.4
Kv4.2 F474A/H478A	0.21 ± 0.04	117.2 ± 3.4	22.6 ± 0.5	0.20 ± 0.04	122.3 ± 4.1	23.9 ± 0.6	0.22 ± 0.04	132.3 ± 5.5	25.0 ± 0.6	0.21 ± 0.04	134.3 ± 4.1	26.1 ± 0.7	0.21 ± 0.04	126.5 ± 3.1	27.6 ± 0.7
Kv4.2 H480A	0.21 ± 0.03	94.6 ± 0.8	18.0 ± 0.2	0.20 ± 0.02	100.0 ± 1.1	18.5 ± 0.2	0.19 ± 0.02	108.6 ± 1.7	19.0 ± 0.3	0.19 ± 0.02	111.8 ± 1.9	19.6 ± 0.3	0.17 ± 0.02	112.7 ± 1.7	21.1 ± 0.4
Kv4.2 L482A/L485A	0.19 ± 0.02	132.8 ± 5.4	25.6 ± 0.8	0.17 ± 0.02	165.6 ± 12.2	28.1 ± 1.1	0.17 ± 0.02	175.2 ± 11.3	29.7 ± 1.1	0.17 ± 0.03	188.1 ± 14.9	31.5 ± 1.4	0.17 ± 0.03	247.8 ± 44.0	35.0 ± 1.9
Kv4.2 H491A/F493A/V494A	0.20 ± 0.05	103.5 ± 6.0	18.9 ± 0.9	0.19 ± 0.04	112.6 ± 5.1	20.0 ± 1.0	0.18 ± 0.04	128.2 ± 6.5	21.1 ± 1.1	0.19 ± 0.04	133.2 ± 5.0	21.9 ± 1.1	0.17 ± 0.04	126.3 ± 6.2	23.7 ± 1.3
**α subunit + KChIP1**															
Kv4.2 WT + KChIP1 WT	0.54 ± 0.08	73.2 ± 2.8	28.3 ± 2.0	0.48 ± 0.07	73.7 ± 4.0	29.6 ± 2.3	0.40 ± 0.07	78.0 ± 7.6	31.1 ± 2.6	0.35 ± 0.07	82.1 ± 11.8	31.6 ± 2.6	0.25 ± 0.06	108.7 ± 31.4	33.4 ± 2.6
Kv4.2 F474A/H478A + KChIP1 WT	0.10 ± 0.02	223.6 ± 19.9	72.5 ± 0.9	0.06 ± 0.01	316.5 ± 53.7	69.8 ± 0.7	0.13 ± 0.03	383.9 ± 81.0	65.9 ± 1.2	0.12 ± 0.04	289.7 ± 74.5	62.7 ± 1.2	n/a	71.3 ± 0.9	n/a
Kv4.2 H480A + KChIP1 WT	0.16 ± 0.03	187.0 ± 27.5	49.2 ± 2.4	0.13 ± 0.02	200.9 ± 25.6	48.8 ± 2.0	0.12 ± 0.02	273.4 ± 26.7	49.0 ± 1.2	0.14 ± 0.03	251.5 ± 15.7	48.4 ± 0.9	n/a	60.0 ± 1.9	n/a
Kv4.2 L482A/L485A + KChIP1 WT	0.27 ± 0.05	193.5 ± 13.7	31.6 ± 1.3	0.27 ± 0.05	286.5 ± 35.7	35.8 ± 1.7	0.33 ± 0.08	444.6 ± 54.3	40.9 ± 2.0	0.36 ± 0.11	482.6 ± 53.4	48.9 ± 3.5	0.25 ± 0.13	164.9 ± 53.2	66.7 ± 6.4
Kv4.2 H491A/F493A/V494A + KChIP1 WT	0.58 ± 0.09	138.3 ± 15.2	52.8 ± 5.8	0.34 ± 0.06	173.6 ± 19.7	65.3 ± 4.1	0.21 ± 0.03	270.9 ± 39.9	72.0 ± 2.4	0.20 ± 0.04	246.6 ± 52.5	74.1 ± 1.6	n/a	91.0 ± 2.6	n/a
**α subunit + DPP6S**															
Kv4.2 WT + DPP6S WT	0.05 ± 0.01	142.0 ± 12.4	10.1 ± 0.6	0.05 ± 0.01	157.4 ± 15.5	10.4 ± 0.6	0.06 ± 0.02	169.4 ± 11.9	10.6 ± 0.6	0.06 ± 0.02	201.4 ± 13.7	11.1 ± 0.6	0.06 ± 0.02	250.8 ± 21.4	11.7 ± 0.6
Kv4.2 T182W + DPP6S WT	0.04 ± 0.01	142.9 ± 11.0	9.7 ± 0.2	0.04 ± 0.01	167.4 ± 14.0	10.1 ± 0.2	0.04 ± 0.01	192.7 ± 26.1	10.3 ± 0.2	0.04 ± 0.01	249.4 ± 49.7	10.8 ± 0.3	0.04 ± 0.01	248.9 ± 32.4	11.5 ± 0.3
Kv4.2 V186W + DPP6S WT	0.05 ± 0.01	134.9 ± 17.0	11.5 ± 0.9	0.05 ± 0.01	154.9 ± 19.9	11.8 ± 0.9	0.05 ± 0.01	181.5 ± 23.2	12.2 ± 0.9	0.05 ± 0.02	234.0 ± 35.4	12.8 ± 1.0	0.05 ± 0.02	261.5 ± 40.9	13.5 ± 1.0
Kv4.2 V190W + DPP6S WT	0.08 ± 0.02	115.4 ± 7.9	15.5 ± 0.9	0.08 ± 0.02	126.1 ± 4.8	16.2 ± 0.9	0.08 ± 0.02	151.8 ± 5.8	17.2 ± 1.0	0.08 ± 0.02	188.7 ± 11.9	18.4 ± 1.2	0.08 ± 0.02	237.4 ± 22.7	20.2 ± 1.4
Kv4.2 F194W + DPP6S WT	0.04 ± 0.01	131.5 ± 9.3	10.5 ± 0.4	0.04 ± 0.01	168.4 ± 12.9	10.9 ± 0.4	0.05 ± 0.01	232.4 ± 19.4	11.2 ± 0.4	0.06 ± 0.01	325.9 ± 36.6	11.6 ± 0.4	0.06 ± 0.01	428.0 ± 54.9	12.3 ± 0.4
Kv4.2 A228W + DPP6S WT	0.06 ± 0.01	109.1 ± 3.0	11.0 ± 0.1	0.06 ± 0.01	118.5 ± 4.4	11.4 ± 0.1	0.06 ± 0.01	133.6 ± 5.4	11.8 ± 0.1	0.06 ± 0.01	155.6 ± 8.5	12.4 ± 0.2	0.07 ± 0.01	169.8 ± 8.3	13.1 ± 0.2
Kv4.2 C231W + DPP6S WT	0.07 ± 0.02	130.6 ± 16.0	18.1 ± 0.8	0.07 ± 0.02	161.2 ± 23.8	19.5 ± 0.9	0.08 ± 0.02	186.7 ± 34.4	20.7 ± 1.1	0.09 ± 0.02	193.0 ± 31.4	22.1 ± 1.3	0.10 ± 0.02	239.9 ± 66.8	24.2 ± 1.6
Kv4.2 A235W + DPP6S WT	0.04 ± 0.01	120.0 ± 5.8	9.9 ± 0.3	0.04 ± 0.01	150.9 ± 7.1	10.3 ± 0.3	0.05 ± 0.01	180.7 ± 9.8	10.5 ± 0.4	0.06 ± 0.01	231.8 ± 14.0	11.1 ± 0.4	0.06 ± 0.01	304.0 ± 30.0	12.0 ± 0.4

Supplementary Table. Inactivation kinetics obtained at 0 to 40 mV.

Inactivation time constants (τ_{slow} and τ_{fast}) and the corresponding amplitude (A_{slow} and A_{fast}) were obtained by fitting the inactivation time course to an exponential function or a sum of two exponentials (n = 8).
