# Supplementary material for: Structural basis of gating modulation of Kv4 channel complexes
Source: Nature. 2021 Sep 22;599(7883):158–64. doi: 10.1038/s41586-021-03935-z (PMC8566240; doi:10.1038/s41586-021-03935-z)
Supplement: Supplementary file 2 — Reporting Summary [file 41586_2021_3935_MOESM2_ESM.pdf]

nature portfolio

Corresponding author(s): Osamu Nureki

Last updated by author(s): Jul 27, 2021

Reporting Summary

Nature Portfolio wishes to improve the reproducibility of the work that we publish. This form provides structure for consistency and transparency in reporting. For further information on Nature Portfolio policies, see our Editorial Policies and the Editorial Policy Checklist.

Statistics

For all statistical analyses, confirm that the following items are present in the figure legend, table legend, main text, or Methods section.

n/a	Confirmed	
☐	☒	The exact sample size (*n*) for each experimental group/condition, given as a discrete number and unit of measurement
☐	☒	A statement on whether measurements were taken from distinct samples or whether the same sample was measured repeatedly
☐	☒	The statistical test(s) used AND whether they are one- or two-sided<br>*Only common tests should be described solely by name; describe more complex techniques in the Methods section.*
☒	☐	A description of all covariates tested
☒	☐	A description of any assumptions or corrections, such as tests of normality and adjustment for multiple comparisons
☐	☒	A full description of the statistical parameters including central tendency (e.g. means) or other basic estimates (e.g. regression coefficient) AND variation (e.g. standard deviation) or associated estimates of uncertainty (e.g. confidence intervals)
☐	☒	For null hypothesis testing, the test statistic (e.g. *F*, *t*, *r*) with confidence intervals, effect sizes, degrees of freedom and *P* value noted<br>*Give P values as exact values whenever suitable.*
☒	☐	For Bayesian analysis, information on the choice of priors and Markov chain Monte Carlo settings
☒	☐	For hierarchical and complex designs, identification of the appropriate level for tests and full reporting of outcomes
☒	☐	Estimates of effect sizes (e.g. Cohen's *d*, Pearson's *r*), indicating how they were calculated

Our web collection on statistics for biologists contains articles on many of the points above.

Software and code

Policy information about availability of computer code

Data collection	EPU (version 1.19), Serial EM (version 3.7.10), Clampex (version 10.7)
Data analysis	RELION (version 3.0 and version 3.1), PHENIX (version 1.19), MOLREP (version 11.7), COOT (version 0.8.9.1), UCSF Chimera (version 1.14), CueMol2 (http://www.cuemol.org/ version 2.2.3.443), HOLE (version 2.2.004), Clampfit (version 10.7), EZR (version 1.54)

For manuscripts utilizing custom algorithms or software that are central to the research but not yet described in published literature, software must be made available to editors and reviewers. We strongly encourage code deposition in a community repository (e.g. GitHub). See the Nature Portfolio guidelines for submitting code & software for further information.

Data

Policy information about availability of data

All manuscripts must include a data availability statement. This statement should provide the following information, where applicable:

- Accession codes, unique identifiers, or web links for publicly available datasets
- A description of any restrictions on data availability
- For clinical datasets or third party data, please ensure that the statement adheres to our policy

The cryo-EM density maps and atomic coordinates have been deposited in the Electron Microscopy Data Bank. The accession codes for the maps are EMD-31010 (Kv4.2-KChIP1-whole (map A)), EMD-31009, (Kv4.2-KChIP1-whole (map A)), EMD-31005 (Kv4.2-KChIP1-TM (map B)) , EMD-31013 (Kv4.2-DPP6S-whole (map E)), EMD-31011 (Kv4.2-DPP6S-TM and Cyto (map F)), EMD-31012 (Kv4.2-DPP6S-TM and EC (map G)), EMD-31019 (Kv4.2-DPP6S-KChIP1-whole (map H)), EMD-31016 (Kv4.2-DPP6S-KChIP1 (TM and Cyto (map I)), EMD-31018 (Kv4.2-DPP6S-KChIP1-TM and EC (map J)), and EMD-31399 (Kv4.2 alone (map X)). The accession codes for the coordinates are 7E84 (Kv4.2-KChIP1-whole), 7E83 (Kv4.2-KChIP1-Cyto), 7E7Z (Kv4.2-KChIP1-TM), 7E8B (Kv4.2-DPP6S-whole), 7E87 (Kv4.2-DPP6S-TM and Cyto),

7E89 (Kv4.2-DPP6S-EC), 7E8H (Kv4.2-DPP6S-KChIP1-whole), 7E8E (Kv4.2-DPP6S-KChIP1-TM and Cyto), 7E8G (Kv4.2-DPP6S-KChIP1-EC), and 7F0J (Kv4.2 alone). For detail, see also Extended Data Table 1, Extended Data Fig. 3, 4, 8, 9.

Field-specific reporting

Please select the one below that is the best fit for your research. If you are not sure, read the appropriate sections before making your selection.

☒ Life sciences ☐ Behavioural & social sciences ☐ Ecological, evolutionary & environmental sciences

For a reference copy of the document with all sections, see nature.com/documents/nr-reporting-summary-flat.pdf

Life sciences study design

All studies must disclose on these points even when the disclosure is negative.

Sample size	No statistical method was used to determine the sample size. For cryo-EM analyses, sample sizes were determined by the availability of microscope time and the number of particles on electron microscopy grids enough to obtain a structure at the reported resolution. For electrophysiological analyses, sample sizes were determined based on the previous reports of this type of study and the reproducibility of results across independent experiments.
Data exclusions	For cryo-EM analyses, particles that did not contribute to improving map quality were excluded following the standard classification procedures in RELION. This is standard practice for structure determination by cryo-EM. For electrophysiological analyses, recordings that contain leak or endogenous currents were excluded. This is standard practice in electrophysiology.
Replication	For cryo-EM analyses, related experiments including FSEC, purification, and SDS-PAGE were reproduced at least two times and structure determination was completed once. For electrophysiological analyses, all data sets were pooled from at least two independent oocyte batches.
Randomization	For cryo-EM analyses, particles were randomly assigned to half-maps for resolution determination following the standard procedures in RELION. For electrophysiological analyses, randomization was not performed since samples were not divided into two or more groups.
Blinding	For cryo-EM analyses, blinding was not applicable since this type of studies does not use group allocation. For electrophysiological analyses, blinding was not applied since it was not technically or practically feasible to do so.

Reporting for specific materials, systems and methods

We require information from authors about some types of materials, experimental systems and methods used in many studies. Here, indicate whether each material, system or method listed is relevant to your study. If you are not sure if a list item applies to your research, read the appropriate section before selecting a response.

Materials & experimental systems

n/a	Involved in the study	
☒	☐	Antibodies
☐	☒	Eukaryotic cell lines
☒	☐	Palaeontology and archaeology
☐	☒	Animals and other organisms
☒	☐	Human research participants
☒	☐	Clinical data
☒	☐	Dual use research of concern

Methods

n/a	Involved in the study	
☒	☐	ChIP-seq
☒	☐	Flow cytometry
☒	☐	MRI-based neuroimaging

Eukaryotic cell lines

Policy information about cell lines

Cell line source(s)	HEK293S GnTI- (ATCC, Cat.#CRL-3022), Sf9 (ATCC, Cat.#CRL-1711)
Authentication	The cell lines listed above were purchased from ATCC Cell lines and no further authentication was performed.
Mycoplasma contamination	Not performed
Commonly misidentified lines (See ICLAC register)	HEK cells are listed in the register but it does not specify which type of HEK strains. Our secondary HEL293S GnTI- cell lines was purchased by from ATCC, where they validated.

Animals and other organisms

Policy information about studies involving animals; ARRIVE guidelines recommended for reporting animal research

Laboratory animals	Adult female Xenopus laevis were used to obtain oocytes.
Wild animals	The study did not involve wild animals.
Field-collected samples	The study did not involve samples collected from the field.
Ethics oversight	All electrophysiological experiments were approved by the Animal Care Committee of Jichi Medical University (Japan) under the protocol no. 18027-03 and were performed following the institutional guidelines.

Note that full information on the approval of the study protocol must also be provided in the manuscript.
